# Annotations

**Published:** 1893-09-23

**Authors:** 


### ANNOTATIONS.

**The New Coachman of the British Association.**

It is announced that the Marquis of Salisbury, K.G., has accepted the offer of the presidency of the British Association for the advancement of science for the year 1894-95. It is somewhat of a new departure that a man who is avowedly not a specialist, but a generalist of the widest possible sympathies, both by necessity and by choice, should have been chosen to occupy a position which hitherto has been looked upon as the throne of the very elect of all the specialists and specialisms. But so it is ordered by destiny. The British Association will be more honoured in its president than the president in the office which he has been called upon to hold. It will be entirely satisfactory to mankind at large to have such convincing proof as this appointment offers that the man is always, and must be greater than the specialist, the specialists themselves being judges. That Lord Salisbury, though not a specialist, is a man of science, the most prejudiced specialist is obliged to admit. How, indeed, can any man of high culture and normal curiosity be other than a man of science in these days —days in which our very puddings are scientifically compounded, and the garden gates of our suburban villas are closed at night by automatic and scientifically constructed porters who require no wages? We are all men of science in such piping times of high scientific progress; we are all for "the advancement of natural knowledge" in every possible way. Every one of us is ready at a moment's notice to put his shoulder to the wheel of the triumphant chariot, and to give it "a long shove and a strong shove" up the steepest hill in order that it may the sooner reach the coveted and still distant top. And we shall all be mighty proud when we see the great Marquis firmly seated on the box-seat next year, and touching up the wheelers with a deft and experienced whip. It is clear that for science, at any rate, "there's a good time coming." We heartily congratulate the British Association on its new coachman.

**Is Vivisection Moral?**

"A MIDDLE-AGED NURSE," whose letter we publish elsewhere, desires to inform the public through our columns that she objects to vivisection on "moral grounds"; and she considers that the question of practical experimentation need not be discussed until the moral quality of vivisection has been decided upon. A good many persons of the highest moral worth hold the same views as does this kindly nurse, and we think it well, therefore, to devote a little space to the subject. First of all: What is morality, and on what do its sanctions rest? There are at least two sets of views prevalent upon this subject. One class of persons hold that morality must be based upon religion. Another class hold that it must be based upon utility. To prove that a thing is moral, say the latter, it is only necessary to prove that it is useful. But at least half the world, at any rate in practice, belongs to this class of utilitarians; and, therefore, by at least half the world, vivisection is held to be highly moral, because it has been proved to be highly useful. There is, then, only the other half of the world to be considered. Now, what is the moral objection which a small portion of the religious world has against vivisection? Is it not this, and this alone, that living animals are put to suffering and pain? And if living animals were not put to suffering and pain by any other means than vivisection, the argument of the "moral" anti-vivisectors would be exceedingly difficult to answer. But, as a matter of fact, "the whole creation," as St. Paul says, "groans and travails in pain together until now." That is to say, from the dawn of creation down to the present time, pain, and severe pain, has prevailed universally throughout the world; and has prevailed because such has always been, and now is, the manifest will of God. It is clear, therefore, that the Creator has no objection to pain as pain. That He reprobates unnecessary pain, or pain inflicted for inadequate objects, we can well believe. But this much at least is indisputable, that the Creator Himself inflicts pain upon helpless animals for adequate objects. It is equally clear that He has given to man the absolute right to do the same for adequate objects. For we are told in the Book of Genesis and elsewhere that the lower animals were given to man for fo[illegible]; and men cannot use them for food without putting them to the severest of all pain, the pain of mortal agony and death. Moreover, the founder of Christianity approved of the killing of animals for food and sacrifices. He Himself ate both fish and meat, and consistently endorsed the profuse slaughter of all kinds of living animals associated with the temple services at Jerusalem. We stand then on this ground, that the whole world, both religious and non-religious, has through all its ages inflicted pain and death on living animals on adequate grounds. The question thus narrows itself to this single inquiry—Is the acquisition of knowledge, which will enable us to relieve pain and to save life throughout the whole world of men and animals, an adequate justification for inflicting pain upon a strictly limited number of animals? In our judgment the justification is ample and complete. There is precisely the same justification for vivisection in search of medical knowledge as there is for the killing of animals for food. In this conclusion we hope our correspondent, and all other reasonable religionists, will entirely coincide.

**More Wicked than Sacrilege.**

A REPORT of shameful wickedness comes to us from Norwood in connection with the local Saturday Hospital Fund. It appears that for some years the collectors of the fund at Norwood have been disappointed with the totals collected, so much so, indeed, that there has often been a difficulty in finding responsible persons to take charge of the collecting boxes. This year it was determined to make an effort to find out whether or not the actual sums collected found their way intact to the central authorities. Accordingly thirteen of the collecting boxes were taken to the house of the Rev. Walter Hobbs, of Salter's Hill, and there opened and the contents counted in the presence of trustworthy witnesses. The amount contained in each box was duly registered, as also the total of the thirteen boxes, amounting to £47 15s. 9d. The boxes were then sealed in the presence of the witnesses, and transmitted through the ordinary local channels to the central authorities of the Saturday Fund. But when they came to be opened it was discovered that the sum of £34 9s. 2d. was the whole of their contents, instead of the £47 15s. 9d. certified by the Rev. Walter Hobbs and other witnesses. Moreover, the sum of £34 9s. 2d. was returned through the local channels as the full amount collected. Mr. Hobbs has, of course, communicated the facts to the central authorities of the Saturday Fund, and also placed the matter in the hands of the police at Scotland Yard. Further developments are awaited. But in the meantime it is not too much to say that any man or woman who robs the sick poor of money raised for their help by self-denying charity commits a crime which is worse than sacrilege, and deserves the sternest punishment which outraged humanity and the law can mete out.

